# Hearing, smell, and cognitive function after cancer treatment

**DOI:** 10.1186/s12885-025-14861-y

**Published:** 2025-10-01

**Authors:** Stavros Potsakis, Juha Tapio Silvola, Ellen Karine Grov

**Affiliations:** 1 Department of Otorhinolaryngology, Akershus University Hospital, Sykehusveien 25, 1474 Akershus, Nordbyhagen Norway; 2 Faculty of Medicine, University of Oslo, Campus Ahus, Akershus, Norway; 3 Faculty of Health and Social Sciences, University of South-Eastern Norway, Drammen, Norway; 4 Faculty of Health Sciences, Oslo Metropolitan University, Oslo, Norway

**Keywords:** Cancer, Platinum-based Chemotherapy, Hearing assessment, Olfactory assessment, Cognitive assessment, Taxane-based chemotherapy

## Abstract

**Objective:**

This study investigates the sensory and cognitive impact of cancer and its treatment, focusing on possible chemotherapy-induced hearing and olfactory impairment, and cognitive function. The primary aim is to evaluate the effectiveness and feasibility of an extended test battery for assessing sensory and cognitive function in cancer patients, providing foundational knowledge for a larger study. A secondary aim is to examine associations between chemotherapy types and sense-neurodegenerative function.

**Design:**

An observational cross-sectional, pilot study evaluated hearing, olfactory function, and cognitive function in first-line chemotherapy patients without prior brain injuries and ototoxic or otological histories. Self-reported outcomes on communication strategies, tinnitus and olfaction were collected. Data analysis applied descriptive statistics with t-tests, and Fisher´s exact tests to compare auditory, olfactory, and cognitive performance between treatment groups.

**Study sample:**

Thirteen cancer survivors (*n* = 13), six (*n* = 6) females and seven (*n* = 7) males who received two different types of chemotherapy.

**Results:**

No significant differences were observed between the chemotherapy groups in audiological and olfactory tests, cognitive assessment, or self-reported outcomes. However, among those receiving platinum-based chemotherapy, participants reported greater use of communication strategies in specific areas.

**Conclusion:**

No significant differences in hearing, olfactory, cognitive, and self-reported outcomes were found when examining cancer patients receiving two different chemotherapy types. The study highlights the need for advanced diagnostic tools to detect hearing, olfactory, and cognitive function in cancer survivors.

**Supplementary Information:**

The online version contains supplementary material available at 10.1186/s12885-025-14861-y.

## Introduction

Cancer treatment provision has improved over the past decades, and there is also an increased survival rate. The life expectancy among people with cancer is increasing, making the consequences of cancer survival increasingly relevant. Statistics from the Cancer Registry of Norway show that 77% experience survival of more than five years [9]. Nevertheless, the survival rate varies significantly and depends on the type of cancer and the patient's response to treatment. The lasting effects after cancer and its treatment have a significant impact on cancer survivors. Cancer and its treatment affect various aspects of life, including, somatic, mental, cognitive, and socioeconomic factors [27].

Several studies have investigated the neuropsychological effects and cognitive decline that follows cancer treatment in cancer survivors [16, 36, 38]. Other studies claim that hearing impairment may be associated with cognitive impairment [23, 45, 53].

Hearing impairment is a well-known comorbidity among patients undergoing chemotherapy [40, 54]. Particularly, platinum-based chemotherapy is associated with an increased risk of ototoxicity, often leading to clinically significant hearing impairment [30, 34, 41]. Conversely, studies examining taxane-based chemotherapy and its potential impact on hearing loss remain limited [55].

In recent studies, Sanchez et al. [41] found that hearing impairment in testicular cancer patients was associated with cognitive impairment, fatigue, depression, and reduced quality of life. It is assumed that the link between hearing impairment and dementia may be related to dysfunction of the auditory cortex, though this hypothesis requires further investigation [25, 51]. In Norway, studies on the side effects of platinum-based chemotherapy in the treatment of testicular cancer have revealed ototoxic damage including hearing impairment and tinnitus [48].

Hearing impairment from platinum-based chemotherapy has substantial effects on daily life, negatively impacting social interaction, work performance and overall well-being. Despite these impacts, follow-up care for chemotherapy-induced hearing impairment remains inadequate, highlighting the need for improved clinical awareness and patient support [34].

Olfactory dysfunction is another prevalent yet under-recognized side effect of chemotherapy. Drareni et al. [13] reported that platinum-based chemotherapy severely affected taste and olfactory perception, leading to altered food experiences and diminished quality of life. Additionally, olfactory loss can occur as a result of brain injuries, cancer, and toxicity [44]. Recent research also shows that olfactory impairment can be an early sign of the development of neurodegenerative diseases such as Alzheimer’s disease (AD) and Lewy body dementia (LBD) [12].

There remains limited research on the long-term effects of chemotherapy on hearing and cognitive function. Platinum-based chemotherapy is thought to have higher toxicity compared to other types of chemotherapy (for example, taxanes) partially due to its prolonged chemical half-life [4]. Regardless of chemotherapy type, studies have shown that cognitive dissonance, often referred to as “chemo brain”, is frequently reported by breast cancer patients undergoing chemotherapy [6]. Similar patterns have been observed among testicular cancer survivors [49].

Specific evaluation methods and suitable measurement tools are essential to identify hearing or olfactory loss, as these tests may give an early indicator of cognitive impairment [29]. It can be assumed that both hearing and olfactory impairment may represent early markers for risk of cognitive impairment.

### Study aims

The primary aim of this study is to evaluate the feasibility of an extended test battery in a clinical setting, comprising assessments of audiological, olfactory, cognitive, and self-reported outcomes among cancer survivors treated with either platinum-based or taxane-based chemotherapy. By identifying methodological strengths and limitations, the study aims to refine testing procedures, laying the foundation for a larger full-scale study in the future. An additional aim is to investigate potential associations between chemotherapy types and sense- and neurodegenerative function. Through these efforts, this research aims to provide valuable insights into the sensory and cognitive effects of chemotherapy, ultimately aiding in the development of improved diagnostic and rehabilitation strategies for cancer survivors.

## Material and methods

### Study design

This study employed an observational, cross-sectional pilot design. The participants, consisting of cancer survivors (*n* = 13), were recruited from the urology, gynecology, and oncology departments of Akershus University Hospital (Akershus, Norway) and had been diagnosed with testicular, bladder, ovarian or breast cancer. Each participant underwent a comprehensive multifunctional test battery during a single session, lasting approximately 1.5 h, with all assessments conducted at a single time point following the completion of chemotherapy and without any follow up evaluations. Participants were scheduled to complete the test battery approximately 6–12 months after completion of chemotherapy. The mean interval between the end of chemotherapy and the post-treatment test battery was 346 days (*SD* = 212), with a median of 291 days. The study received ethical approval from the Norwegian Regional Committee for Medical and Health Research Ethics (REC) (case no: 230844). Additionally, all sensitive personal data were managed in compliance with the guidelines set by the Data Protections Officer (DPO) (case no: 2021_84) at Akershus University Hospital, ensuring adherence to ethical and legal data handling standards.

### Participants and inclusion criteria

Participants were selected according to predefined inclusion criteria to ensure a targeted and relevant study sample. The mean age of the participants was 58 years (*SD* = 11.9), with an age range of 18 to 65 (Table 1). This study included this age range to minimize confounding variables associated with age-related hearing loss [32]. All participants had received either platinum-based or paclitaxel (taxane-based chemotherapy) following a first-time cancer diagnosis and included both male and female patients. Individuals were excluded if they had previously undergone hormone, immunotherapy or radiation therapy, or received a combination of chemotherapy types. Additional exclusion criteria included a documented history of cognitive impairment, diagnosed neurological disorders (e.g., traumatic brain injury, brain metastases), head and neck cancer, progressive hereditary hearing impairment, chronic ear disease, or prior ear surgery. Pre-existing cognitive difficulties were identified based on available medical records and self-reported medical histories prior to the onset of chemotherapy. However, formal baseline cognitive assessments were not conducted before chemotherapy initiation. Similarly, no formal pre-treatment audiological or olfactory testing was performed; thus, exclusion relied on patient history and clinical documentation. Additional exclusion criteria included the use of hearing aids, prior exposure to ototoxic medications, untreated cardiac or pulmonary conditions, mental health disorders impacting cognitive functioning, and substance abuse history. Furthermore, demographic and clinical data, including marital status, educational level, residential area, chemotherapy details (platinum- or taxane-based) were collected.

**Table 1:** Sociodemographic characteristics and clinical features for 13 cancer survivors

Characteristics	Total (*n* = 13)	Men (*n* = 7)	Women (*n* = 6)	*p*-value
Gender (%)	100.0%	53.8%	46.2%	
Age, mean (*SD*) (Years)	57.77 (11.19)	55.86 (14.72)	60.00 (5.40)	0.53
Level of Education (*n*)				0.17
- Less than 13 years	6	2	4	
- More than 13 years	7	5	2	
Marital Status (*n*)				0.42
- Married/Partner	10	6	4	
-Divorced/Separated/Single	3	1	2	
Place of Residence (*n*)				0.80
- Urban—Municipality	7	4	3	
- Rural—Municipality	6	3	3	
Type of Chemotherapy (*n*)				0.002
- Platinum-based	8	7	1	
- Taxane-based	5	0	5	

### Audiological assessment

A bilateral *otoscopic examination* was conducted to assess for any obstructions or abnormalities, thereby confirming normal otologic conditions prior to the audiological evaluation. *Tympanometry (226 Hz)* was then conducted with a handheld Interacoustics Titan (Diatec Diagnostics) tympanometer to confirm normal tympanic membrane and middle ear function.

*Pure Tone Audiometry* was performed in a soundproof room (IAC 120A-10) using an Otometrics- Madsen® A450 PC-based audiometer (Natus Medical), with circumaural headphones (RADIOEAR—DD450) for air-conduction threshold testing, including extended high-frequency audiometry capable of testing frequencies up to 16 kHz, compliant with the IEC-60645–1 and ANSI S3.6 standards. Bone-conduction thresholds were assessed using a bone-conductor (B71W). Air- and bone-conduction hearing thresholds were measured across ten octave test frequencies. Air-conduction thresholds were assessed from 0.125 kHz to 8 kHz, while bone-conduction thresholds were measured at selected frequencies between 2.5 kHz and 4 kHz. The pure-tone average (PTA4) from the air-conductions thresholds was calculated by determining the mean of the thresholds at 0.5, 1, 2 and 4 kHz, following the definition of the World Health Organization. Additionally, *Extended High-Frequency Pure Tone Audiometry* (9—12.5 kHz) was conducted to assess hearing sensitivity at higher frequencies, which may help identify early signs of hearing loss or ototoxicity [7].

*Speech Audiometry* was performed using a Norwegian monosyllabic word list to determine PBmax. Testing began at PTA4 + 25 dB HL and continued in 10 dB increments until the maximum word recognition score was reached [15].

*Hearing-in-Noise Test* (HINT; [33]) was used to evaluate speech perception in noise. The Norwegian HINT was performed in a soundproof room (IAC 120A-10) in a free field setting. Participants were presented with sentences (12 corresponding lists with 20 everyday-life sentences each, containing four to eight words) in both quiet and noisy environments [31]. The sentence order was randomized within each list, and participants repeated the sentences or segments they could discern. The average threshold for speech perception, also known as the Speech Reception Threshold (SRT), was calculated based on the responses to the 20 sentences [39, 47].

Testing was conducted using two Genelec 8010AOM active monitors, each producing low frequencies (LF) at 3 levels and high frequencies (HF) at 75 levels. Participants sat 1 m from each speaker, with a ceiling-mounted laser pointer ensuring precise position at the center of the participant´s head. Speech signals were always presented from the front speaker (0 $^\circ$ azimuth). In quiet conditions, sentences were presented at 20 dBA. Noise was set at 65 dBA for three test conditions: noise from the front (NF), noise from the right (NR) and noise from the left (NL). The standard testing protocol adjusted levels in 4 dB increments for the first four sentences and in 2 dB increments for the remaining 16 sentences. Speech levels were automatically modified depending on correct or incorrect response. [31].

*Transient Evoked Otoacoustic Emissions (TEOAE) and Distortion Product Otoacoustic Emissions (DPOAE)* measurements were recorded and analysed using the same handheld Interacoustics Titan tympanometer equipped with OAE-protocols. Both measurements aimed to correlate with the auditory thresholds obtained from the pure tone audiometry [50]. TEOAE responses were recorded using click stimuli at 80 dB SPL, with response collection occurring between 4 and 12.3 ms and filtered between 0 and 6000 Hz. Amplitude, response reproducibility, noise floor and signal-to-noise ratio (SNR) were analyzed across the overall frequency range and of ½—octave bands with standard frequencies 1, 1.5, 2, 3 and 4 kHz for both ears [5]. For DPOAE measurements, two-tone stimuli were used with a fixed frequency ratio of f1 to f2 (f1/f2 = 1.22) and intensity levels of 65 dB SPL for L1 and 55 dB SPL for L2. The signal amplitudes were measured at 2f1–f2 frequencies across f2 frequencies ranging from 500 to 10,000 Hz for both ears [35]. For both TEOAE and DPOAE measurements, a diagnostic protocol was used. A pass required detectable emissions at all tested frequencies in both ears, based on predefined signal-to-noise ratio thresholds.

### Olfactory assessment

Olfactory function was assessed using a combination of sniffing sticks and a structured questionnaire. The Sniffing Sticks (Burghardt®, Wedel, Germany) consist of 12 scented felt-tip pens, each containing a distinct odorant. Participants were asked to identify each scent and select the correct answer from four options presented on a Norwegian-language based answer sheet. The Smelling Questionnaire (TSQ) was specifically developed for the purpose of this study to assess participants` olfactory function and potential challenges in differentiating between various odors. The questionnaire comprised 10 questions in Norwegian, addressing abilities in scent recognition, the duration of any olfactory issues, and the extent to which these issues affect their daily lives. Additionally, the questionnaire examines related nasal symptoms, such as congestion or rhinorrhea, as well as other associated discomforts. As this instrument was newly developed for this study, it has not undergone psychometric validation or been piloted with in a non-cancer population (Appendix A).

### Cognitive function assessment

Cognitive function was evaluated using the Norwegian version of Montreal Cognitive Assessment (MoCA; Appendix B) [11]. This cognitive screening measure is reliable for evaluating various aspects of cognitive function, including attention, memory, language and visual recognition of images and shapes [22]. To assess planning and executive function, the Trail Making Test (TMT) parts A and B were used [52].

### Self-reported outcomes

Self-reported outcomes were assessed by using *the Communication Strategies Scale (CSS)* and the *Tinnitus Handicap Inventory (THI-NOR)* questionnaires, both adapted into Norwegian and psychometrically tested. The CSS-questionnaire (Appendix C) assesses the communication strategies employed by individuals experiencing hearing difficulties in various everyday situations. It provides insight into participant´s abilities to adapt and manage the communication difficulties they encounter in everyday life [20]. The questionnaire consists of 25 items, each rated on a five-point Likert scale ranging from “never” to “almost always.” The responses are scored from 1 to 5, respectively, with higher scores reflecting more frequent use of communication strategies. The CSS covers three subscales: maladaptive behaviors, verbal strategies, and non-verbal strategies. This structure enables a detailed evaluation of how individuals cope with communication difficulties, offering a comprehensive picture of their behavioral responses and adaptive techniques.

The THI-NOR evaluates the impact of tinnitus on daily life and quality of life, offering a measure of the burden imposed by tinnitus [18]. The questionnaire consists of 25 questions with response options of “yes”, “sometimes”, and “no”, corresponding to scores of 4, 2, 0 points, respectively. The total score ranges from 0 to 100, with higher scores indicating greater tinnitus-related distress (Appendix D). The THI-NOR assesses three domains: functional, emotional, and catastrophic impacts of tinnitus, providing a comprehensive assessment of the participants` experience.

### Procedure

Data collection for clinical and cognitive assessments was conducted in an approved examination room at the otorhinolaryngology department at Akershus University Hospital. Participants received detailed study information and provided written informed consent before testing (Appendix E). A general anamnesis was obtained first, followed by the completion of self-report questionnaires, audiological assessment, olfactory assessment, and cognitive evaluations. The test order was fixed, with participants completing the assessments in the same sequence. While this fixed order may introduce potential fatigue or order effects, all tests were relatively brief and structured to minimize such biases. All questionnaires were completed in a quiet, interruption-free environment within the same department. Participants received comprehensive information about each test and were given the opportunity to ask questions both before and during testing. Breaks were offered as needed. Several participants reported experiencing a degree of performance anxiety, particularly with the MoCA. To mitigate this, they were offered a review of their test results at the end of the sessions, along with an opportunity to ask questions and reflect on the testing process. This approach was grounded in careful ethical considerations, recognizing the importance of supporting participants` sense of competence, and addressing any concerns they might have. For those who found the tests challenging, an option for follow-up support was available (preparedness described in the information and consent form).

### Data analysis

Statistical analyses were performed using IBM SPSS (Version 29.0.0) to assess baseline values and determine statistical significance across variables. Descriptive statistics, including means, standard deviations, frequencies, and percentages were used to outline the baseline characteristics of the sample. For group comparisons between platinum-based (Chemo1 (P)) and taxane-based (Chemo 2 (T)) treatments, independent sample t-test and Fisher’s exact tests (for n < 5) were applied due to the small sample size, while chi-square test (χ^2^-tests) were used for categorical variables. Hearing thresholds for standard frequencies and high frequencies (0.125–12.5 kHz) in both ears were measured, and an average threshold value from each frequency and PTA4 was calculated to provide a single value representing binaural hearing sensitivity. Hearing thresholds were then compared between the two chemotherapy groups to examine the mean differences in auditory sensitivity. No age correction was applied when analyzing hearing thresholds for this particular study. Hearing in noise performance from the HINT was evaluated by comparing the groups` average speech reception threshold in quiet and the level of signal-to-noise ratio (dBSNR) in the settings including speech in background noise (NF, NR and NL).

The analysis for the TEOAE and DPOAE measurements focused on the average outcomes for each frequency, with results categorized as either “pass” or “no pass”, This analysis was conducted separately for each group and an overall performance measure was obtained by calculating the average pass rate at each frequency. For the MoCA, overall scores as well as specific domain scores from both chemotherapy groups were compared. For TMT A and B, scores were compared between the groups. For the CSS-questionnaire, the analysis was obtained by comparing the mean response on each item between the two groups. For the THI-NOR, mean total scores were compared between the two groups. Python (Version 3.13.1) was used for figure generation and data visualization.

## Results

### Sociodemographic differences

Among 13 participants, 53.8% were men with a mean age of 56 (*SD* = 14.72) years and 46.2% were women with a mean age of 60 (*SD* = 5.40) years (Table 1). The majority of participants were married or cohabiting (10 in total). Educational attainment varied by gender, with more men (*n* = 5) having more than 13 years of education compared to women (*n* = 2). Residence was evenly distributed between urban and rural municipalities. A statistically significant difference was found in the type of chemotherapy (*p* = 0.002) received, with platinum-based treatment being more common among men, while taxane-based treatment was exclusively administered to women.

### Differences in audiological measures

#### Pure Tone Audiometry and PTA4 results

**Fig. 1:**
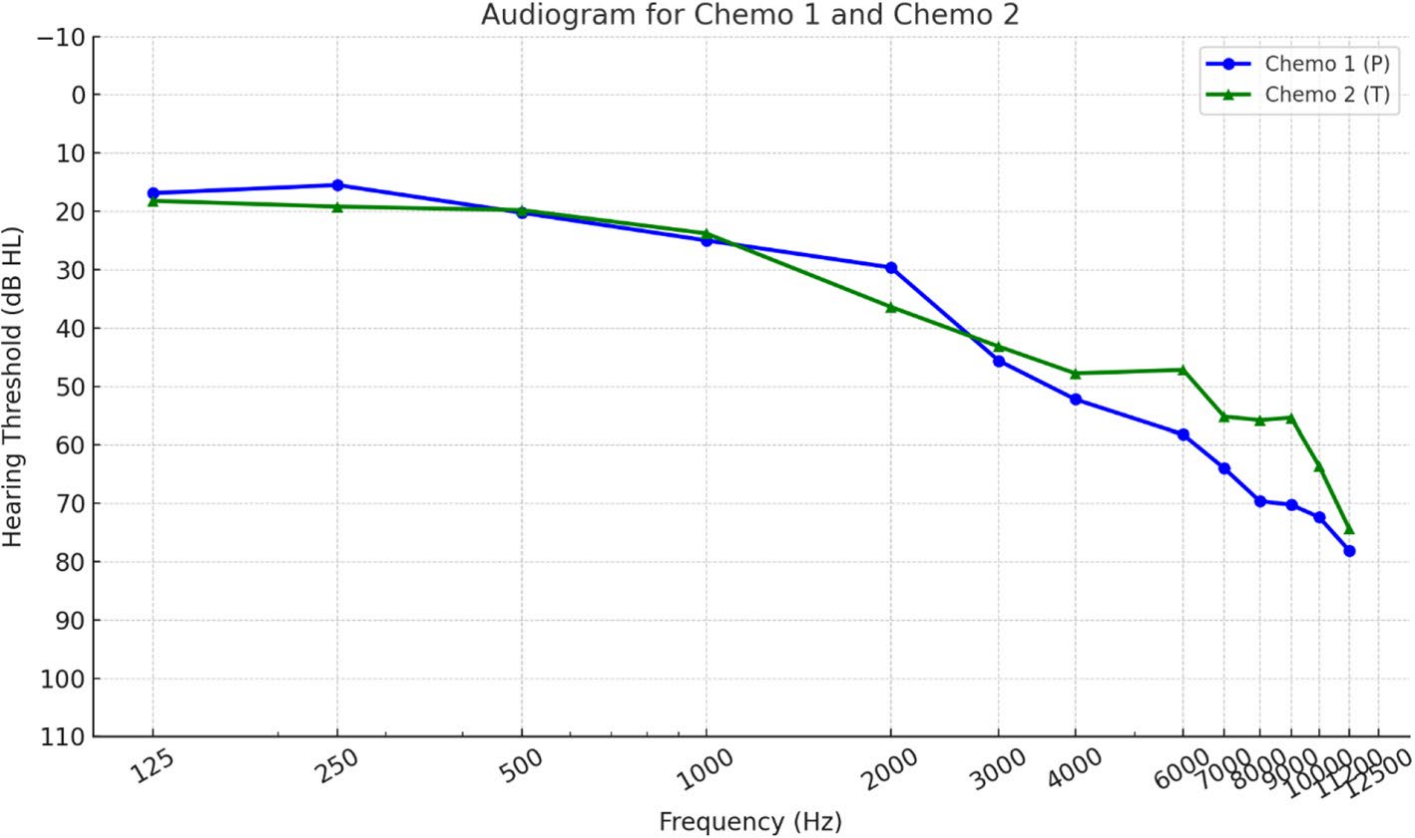
Differences in pure tone audiometry (0.12—12.5 kHz) between Chemo 1(P) and Chemo 2 (T)

Pure tone audiometry results across standard and high frequencies (125 Hz to 12,500 Hz), revealed no significant differences between the Chemo 1 (P) and Chemo 2 (T) groups. Hearing thresholds were comparable, with all *p*-values exceeding 0.05 (Table 2, Fig. 1).

**Table 2:** Differences in pure tone audiometry and high-frequency audiometry between Chemo 1(P) and Chemo 2 (T)

Frequency (Hz)	Sample (*n* = 13) Mean (*SD*)	Chemo 1 (P) Mean (*SD*)	Chemo 2 (T) Mean (*SD*)	*p*-value	CI 95% (Lower, Upper)
125 Hz	17.38 (10.28)	16.88 (9.91)	18.20 (12.01)	0.83	−14.8, 12.12
250 Hz	16.92 (14.68)	15.50 (14.83)	19.20 (15.85)	0.68	−22.78, 15.38
500 Hz	20.08 (13.30)	20.25 (15.84)	19.80 (9.58)	0.96	−16.98, 17.88
1000 Hz	24.54 (14.32)	25.00 (18.32)	23.80 (5.12)	0.89	−17.54, 19.94
2000 Hz	32.23 (15.36)	29.63 (18.52)	36.40 (8.53)	0.46	−26.40, 12.85
3000 Hz	44.69 (21.03)	45.63 (24.70)	43.20 (15.94)	0.85	−25.09, 29.93
4000 Hz	50.54 (23.11)	52.25 (28.24)	47.80 (13.81)	0.75	−25.69, 34.59
6000 Hz	54.00 (20.59)	58.25 (22.05)	47.20 (18.09)	0.37	−13.55, 36.45
8000 Hz	60.62 (22.93)	64.00 (24.40)	55.20 (21.81)	0.52	−20.68, 38.77
9000 Hz	64.31 (23.27)	69.63 (22.01)	55.80 (25.09)	0.32	−15.25, 42.91
10,000 Hz	64.54 (26.14)	70.25 (27.11)	55.40 (24.37)	0.34	−17.96, 47.66
11,500 Hz	69.00 (24.20)	72.38 (28.49)	63.60 (16.65)	0.55	−22.40, 39.95
12,500 Hz	76.69 (19.28)	78.13 (24.01)	74.40 (9.74)	0.75	−21.42, 28.87

Similarly, the PTA4 score, representing the average thresholds at the conventional speech frequencies (500, 1000, 2000, and 4000 Hz), showed no significant group differences for both ears combined (Table 3). The mean PTA4 scores were 31.63 dB (*SD* = 17.93) for Chemo 1(P) and 31.60 dB (*SD* = 5.68) for Chemo 2 (T), with a *p*-values at 1.0. No age correction was applied in the analysis of hearing thresholds, as the absolute results revealed no significant differences in age between the chemotherapy groups. No difference in age distribution was found between the groups. Therefore, age is unlikely to have confounded the comparison of hearing outcomes between the groups.

**Table 3:** Differences in PTA4 between Audiometry between Chemo 1(P) and Chemo 2 (T)

PTA4 Test (Frequency)	Sample (*n* = 13) Mean (*SD*)	Chemo 1 (P) Mean (*SD*)	Chemo 2 (T) Mean (*SD*)	*p*-value	CI 95% (Lower, Upper)
PTA4_Right	31.54 (13.28)	31.88 (16.15)	31.00 (8.46)	0.91	−16.51, 18.26
PTA4_Left	31.77 (15.62)	31.38 (20.09)	32.40 (4.98)	0.91	−21.48, 19.43
PTA4_Both	31.62 (14.08)	31.63 (17.93)	31.60 (5.68)	1.0	−18.43, 18.48

#### TEOAE and DPOAE tests

Transient Evoked Otoacoustic Emissions (TEOAE) and Distortion Product Otoacoustic Emissions (DPOAE) results across frequencies were non-significant (see Table H 1,Table H 2 in Appendix H).

#### Speech audiometry with monosyllabic words

Speech audiometry scores for both left and right ear were collected across the two groups. For the left ear, the mean score was 47.33 dBHL (*SD* = 23.59) for Chemo 1 (P) and, 60.00 dBHL (*SD* = 11.73) for Chemo 2 (T), with a *p*-value of 0.31. For the right ear the mean score was 50.00 dBHL (*SD* = 20.98) for Chemo 1 (P) and 55.00 dB HL (*SD* = 9.35) for Chemo 2 (T), with a *p*-value of 0.64. The results (Table H 3) show no significant differences in speech audiometry score between the left and right ear or between the chemotherapy groups.

#### Hearing in noise test

The HINT evaluated hearing thresholds in quiet and noisy conditions. The mean threshold and standard deviation for both groups are illustrated in Table 4. No significant differences were observed between the groups when comparing thresholds in quiet conditions (dBA) and the signal-to-noise ratios (dBSNR) in different noise directions (NF, NL, and NR). All *p*-values exceeded 0.05, and 95% CI included zero in all test conditions, suggesting no notable differences in hearing performance between Chemo 1 (P) and Chemo 2 (T) group (Table 4).

**Table 4:** Differences in Hearing in Noise Test (HINT) between Chemo 1 (P) and Chemo 2 (T)

HINT	Sample (n = 13) Mean (*SD*)	Chemo 1 (P) Mean (*SD*)	Chemo 2 (T) Mean (*SD*)	*p*-value	CI 95% (Lower, Upper)
Quiet (dBA)	33.48 (11.37)	33.86 (14.27)	32.88 (5.55)	0.87	−13.90, 15.87
Quiet Score (%)	71.59 (12.01)	68.26 (14.27)	76.92 (4.35)	0.22	−23.31, 6.01
Noise Front (dB SNR)	−1.35 (2.09)	−0.81 (2.37)	−2.20 (1.33)	0.26	−1.19, 3.96
Noise Left (dB SNR)	−4.45 (3.19)	−3.51 (3.57)	−5.96 (1.94)	0.19	−1.41, 6.31
Noise Right (dB SNR)	−5.46(3.01)	−5.45 (3.21)	−5.48 (3.04)	0.99	−3.92, 3.98

### Differences in olfactory measurements

#### Sniffing sticks results

The olfactory assessment using Sniffing Sticks revealed no significant differences between the two groups (Chemo1 (P) and Chemo 2 (T)) suggesting comparable odor detection abilities. The error rates in odor recognition were similar, further supporting similar olfactory function. No particular scent stick proved to be especially difficult or easy to identify (Table H 4).

### Differences in cognitive measurements

#### MoCA and TMT A and B results

The Montreal Cognitive Assessment (MoCA) tested cognitive function. A non-significant trend was observed in the visuospatial/executive function (*p* = 0.08) between groups (Table 5); the results revealed that Chemo 1 (P) scored slightly higher. Other cognitive domains, including attention, language, and delayed recall, showed no significant differences between groups. The overall MoCA Total Score difference was also non-significant (*p* = 0.65) between the groups, the mean score was 24.2 (*SD* = 2.55), which is below the normal threshold (normal ≥ 26/30), indicating that participants, on average, scored lower than the expected range for normal cognitive performance. There was no significant difference in the time taken to complete TMT A (*p* = 0.66) and B (*p* = 0.43) between the two groups. This indicates that their performance speed, which reflects processing speed, attention, and executive functioning, was comparable.

**Table 5:** Differences in objective measures of cognitive function between Chemo 1 (P) and Chemo 2 (T)

MoCA Test	Sample (*n* = 13) Mean (*SD*)	Chemo 1 (P) Mean (*SD*)	Chemo 2 (T) Mean (*SD*)	*p*-value	CI 95% (Lower, Upper)
Visuospatial/Executive	4.38 (0.96)	4.75 (0.46)	3.80 (1.30)	0.08	−0.14, 2.04
Naming	3.00 (0.00)	3.00 (0.00)	3.00 (0.00)	-	-
Attention	4.92 (1.38)	5.13 (1.46)	4.60 (1.34)	0.53	−1.25, 2.30
Language	2.54 (0.78)	2.50 (0.93)	2.60 (0.55)	0.83	−1.12, 0.92
Abstraction	1.77 (0.44)	1.75 (0.46)	1.80 (0.45)	0.85	−0.62, 0.52
Delayed Recall	1.62 (1.26)	1.38 (1.30)	2.00 (1.22)	0.41	−2.22, 0.97
Orientation	6.00 (0.00)	6.00 (0.00)	6.00 (0.00)	-	-
MoCA Total Score	24.23 (2.55)	24.50 (2.67)	23.80 (2.59)	0.65	−2.62, 4.02
Trail Making Test A	38.15 (11.40)	37.00 (10.80)	40.00 (13.38)	0.66	−17.81, 11.81
Trail Making Test B	103.25 (38.43)	112.40 (39.68)	88.00 (38.22)	0.43	−45.65, 94.45

### Differences in self-reported outcomes

#### CSS questionnaire results

In the Self-Reported CSS – Questionnaire, significant differences were observed in the following items: "Position for Hearing" (95% CI [0.02, 3.08], *p* = 0.02), "Positioning" (95% CI [0.02, 3.08], *p* = 0.05), and "Focus" (95% CI [0.50, 2.85], *p* = 0.01), suggesting that participants in the Chemo 1 (P) group more frequently used communication strategies, particularly in selecting optimal positioning for better hearing and focus. Other items showed no significant differences, indicating similar self-reported communication behaviors across groups (Table H 5). Among the sample (*n* = 13), participants demonstrated frequent use of active communication strategies, particularly "Repeat Part" (Q11, Mean = 3.61, *SD* = 1.50), "Ask for Repeat" (Q13, Mean = 3.76, *SD* = 1.36), and "Focus" (Q23, Mean = 3.23, *SD* = 1.24).

#### Tinnitus handicap inventory (THI-NOR) score

The Tinnitus Handicap Inventory (THI-NOR) showed no significant differences in tinnitus-related impact between the two groups, with the 95% confidence interval including zero. For the total sample (*n* = 13), the mean THI-NOR score was 25.23 (*SD* = 21.86). The Chemo 1 (P) group had a mean score of 28.25 (*SD* = 21.82), while the Chemo 2 (T) group had a mean score of 20.40 (*SD* = 23.51), resulting in a *p*-value of 0.55, further confirming no significant difference. Individual item responses also showed no significant variation between groups, suggesting comparable levels of tinnitus-related handicap (Table H 6).

#### The smelling questionnaire (TSQ)

The TSQ results table highlights comparisons of mean scores between Chemo 1 and Chemo 2 groups across questions about olfactory performance. No significant differences (*p*-value > 0.05) were observed for any question, indicating similar results between the treatments (Table H 7).

## Discussion

Previous studies have explored the relationship between cognitive function and cancer treatment among cancer survivors, demonstrating a notable association between chemotherapy and cognitive impairment [16, 36, 38]. Additionally, hearing impairment and olfactory dysfunction are well-documented comorbidities following cancer treatment [13, 14, 40, 48, 54]. Sensory deficits, such as hearing and olfactory impairment, have also been linked to cognitive decline and neurodegenerative diseases [23, 45, 53]. While chemotherapy has been widely implicated in cognitive impairment among cancer survivors, it is increasingly recognized that cancer-related cognitive impairment likely results from a multifactorial etiology. Factors such as the biological effects of the cancer itself, systemic inflammation, psychological distress (e.g., anxiety, depression), cancer-related fatigue, hormonal changes, and the cumulative burden of multiple treatments (e.g., surgery, radiation therapy) may all contribute to cognitive changes [2, 24].

Despite the multifactorial nature of these impairments, chemotherapy remains a central factor of interest, particularly regarding its known neurotoxic and ototoxic effects. Platinum-based chemotherapy has a well-documented risk of neuro- and ototoxicity, leading to both hearing impairment and olfactory dysfunction [13, 17]. In contrast, taxane-based chemotherapy has not been consistently associated with hearing impairment, though evidence suggests a stronger link with olfactory dysfunction [8, 26, 55].

The primary aim of this study was to evaluate the feasibility of our extended test battery in a clinical setting, comprising audiological, olfactory, cognitive assessments and self-reported outcomes in cancer survivors treated with either platinum-based (Chemo 1(P)) or taxane-based (Chemo 2 (T)) chemotherapy. An additional aim was to investigate potential difference between chemotherapy types and sense- and neurodegenerative function. Notably, few comparable studies exist. Xuan et al. [55] highlight the limited research on taxane-based ototoxicity, referencing Atas et al. [3] who observed paclitaxel-induced cochlear damage in mice, leading to mild to moderate sensorineural hearing loss without sensory cell loss. Another study involving 103 cancer patients treated with taxanes (paclitaxel or docetaxel) found no significant audiovestibular side effects, with sensorineural hearing loss observed in only 1.9% of cases [42]. In contrast, findings by Cheung et al. [10] indicate that hearing loss and tinnitus are prevalent among cancer survivors regardless of chemotherapy regimen, suggesting the involvement of shared neurotoxic mechanisms across platinum- and taxane-based treatments.

Our findings revealed no significant differences between the two chemotherapy groups across all measurements in the test battery. This suggests that both chemotherapy treatments exert comparable effects on assessed parameters. Although no significant differences were found, the observed non-significant trends suggest that further research with a larger sample size is needed to determine whether taxanes might exhibit greater ototoxicity than previously recognized.

Hearing assessments, including pure tone audiometry with extended high frequency measurement, speech audiometry, Hearing in Noise Test (HINT), and otoacoustic emission (TEOAE and DPOAE), revealed no significant differences between the two chemotherapy groups. Hearing thresholds across standard and high frequencies, as well as speech-related thresholds, were comparable between the two groups. However, a slight non-significant difference was observed in the high-frequency range (6 kHz–11.5 kHz), where thresholds for Chemo 1 (P) were approximately 10–20 dB worse compared to Chemo 2 (T) (Fig. 1). Given the expectation that the test battery would detect treatment-related differences, these findings raise the possibility that the sample size was insufficient to identify significant effects or that taxanes may be more ototoxic than previously believed.

TEOAE and DPOAE measurements in both ears revealed mostly absent responses at frequencies above 2 kHz, consistent with pure-tone audiometry findings showing a significant decline in hearing thresholds beyond 3 kHz. This correlation between the absence of otoacoustic emissions and elevated hearing thresholds in higher frequencies suggests cochlear dysfunction, likely affecting the outer hair cell activity [43]. Previous studies have demonstrated the utility of TEOAE and DPOAE as objective tools for validating pure-tone audiometry findings and detecting cochlear dysfunction, particularly in higher frequency ranges [21, 37, 43]. Building on prior suggestions from Knight et al. [28], future research could further evaluate the utility of these tests as early indicators of chemotherapy-induced hearing impairment.

No significant difference was found between chemotherapy groups in speech perception measured by HINT in quiet or noisy conditions. However, some participants in both groups exhibited severe difficulties in both conditions, suggesting that individual patients may experience varying degrees of speech perception challenges. Compared to Skalleberg et al. [47], no significant differences in speech perception under quiet (Q) conditions were observed. However, in noisy conditions (NF, NR, NL), the participants in the present study performed worse. This discrepancy may be attributable to differences in pure-tone audiometry thresholds, as participants in Skalleberg et al. [47] exhibited better PTA4 scores, potentially contributing to superior speech perception in noise. Understanding speech in noisy environments is a well-documented challenge for individuals with hearing impairment. Studies indicate that speech perception tends to decline with age, independent of hearing thresholds, likely due to age-related reductions in cognitive processing abilities [1, 17].

Olfactory assessment revealed no significant differences between the two groups in their performance on the Sniffing Stick test, indicating comparable olfactory odor detection abilities. The distribution of errors in odor recognition was similar, reinforcing the conclusion of equivalent olfactory performance. Notably, no specific scent proved particularly challenging or easy for participants to identify. Such data were not analysed as they were not central to the research question. However, analysing specific scent sticks might provide deeper insights or reveal patterns, which could be valuable for discussion or future studies. The variation in the distribution of errors suggests that olfactory recognition ability was not systematically linked to any single odor stimulus. This suggests that individual differences in odor perception were the main factors influencing the test outcomes, rather than the properties of specific scent. Our results suggest that taxanes can also be more toxic to the olfactory function than previously assumed. For the TSQ, non-significant differences were observed between the two groups in scent recognition, olfactory issue duration, or daily life. However, the lack of psychometric validation of the TSQ may affect the reliability of the results.

For the cognitive assessment, the MoCA and TMT A and B found no significant differences between the two groups. A non-significant trend was observed in visuospatial/executive function where Chemo 1 (P) had a higher score than Chemo 2 (T). Other cognitive domains showed no notable differences. The mean total score was 24.20 (*SD* = 2.55), which is below the normal threshold (normal ≥ 26/30). Notably, 9 out of 13 participants scored below the normal cutoff, indicating generally poor cognitive performance after chemotherapy. For the TMT A & B, no significant differences were found in completion times. All participants successfully completed part A within the 100-s time limit. In part B, five participants failed to complete the test due to sequencing errors, though none exceeded the 300-s time limit. These results suggest comparable processing speed and executive functioning between the groups. The errors in TMT B highlight difficulties in task complexity, warranting further investigation. Educational level did not appear to significantly impact cognitive performance in this study.

In self-reported outcomes, the CSS revealed significant differences in three items (“Position for Hearing”, “Positioning”, and “Focus”), suggesting that participants in the Chemo 1 (P) group relied more heavily on physical and cognitive strategies to manage communication difficulties. No significant differences were found in other items from the CSS- questionnaire. The sample (n = 13) demonstrated frequent use of active communication strategies, particularly asking for repetition, repeating conversation segments, focusing attention, and positioning themselves strategically. These proactive behaviours align with Heine et al. [19], who identified repetition as the most commonly reported strategy.

The THI showed no significant difference in tinnitus-related impact between the two groups. Specifically, six participants reported a slight or no handicap, three participants reported a 'mild handicap', and four participants reported a 'moderate handicap'. These findings indicate a low to moderate tinnitus burden with no significant group differences. However, the small sample size limits generalizability, and further research is needed to confirm these findings.

In conclusion, there were no significant differences between the two chemotherapy regimens (platinum-based and taxane-based), in terms of their impacts across sensory function, cognitive performance, and self-reported outcomes. However, a statistically significant difference was found in the distribution of chemotherapy types based on gender with platinum-based treatments more frequently administered to men and, taxane-based treatments exclusively given to women. This difference in treatment allocation likely reflects clinical practices, where treatment decisions are influenced by factors primarily based on cancer type. It also reflects patient characteristics or medical considerations based on cancer severity or stage and other comorbidities [46]. It is important to note that while this study found no significant differences between the two chemotherapy types, the small size may limit the ability to detect subtle variations. Our pilot study shows that this extended test battery can be used in clinical practice, and it is important to test it with larger study population. The small difference between the groups indicates that relatively large study groups are needed to achieve sufficient statistical power. Future studies should aim to include larger and more diverse sample groups to minimize bias and better assess potential differences between the two chemotherapy regimens. That will help ensure that findings can be generalized across broader populations.

### Strengths and limitations

A notable strength of this pilot study is the integration of audiological and olfactory assessments with cognitive measurements and self-reported outcomes. This comprehensive approach provides a multifaceted understanding of the potential impacts of chemotherapy on sensory and cognitive functions. Additionally, the study’s strict inclusion criteria contribute to the reliability of its findings by minimizing confounding variables and may also have contributed to recruitment challenges. However, the primary limitation of this study is the small sample size (n = 13), which restricts the generalizability of the findings and reduces statistical power to detect subtle differences between the chemotherapy groups.

Furthermore, although participants with documented pre-existing cognitive impairments or sensory deficits were excluded based on medical history, no formal baseline assessments of cognitive, hearing, or olfactory functions were conducted prior to chemotherapy. This limitation restricts causal inferences regarding chemotherapy-induced changes. Additionally, the study did not correct for undiagnosed or subclinical hearing loss, including age-related or high-frequency deficits, which may have introduced confounding variability and influenced hearing-related outcomes. Another important limitation concerns cancer type and the timing and dosage of chemotherapy. The timing of assessments relative to the last chemotherapy session was not standardized across participants, and detailed data regarding the number of chemotherapy sessions and cumulative dosage were not systematically collected. This limits the ability to evaluate potential dose–response relationships or the influence of recovery time on sensory and cognitive outcomes. Future studies should ensure detailed documentation of chemotherapy regimens and time elapsed since treatment completion, as these factors may significantly impact the results. In addition, the expected duration of chemotherapy’s influence on sensory and cognitive functions was not addressed and may vary based on individual factors such as treatment intensity, cancer type, and patient age. Future studies should examine how the duration of treatment effects correlates with recovery trajectories and functional outcomes.

Finally, the use of a novel olfactory questionnaire (TSQ) without psychometric validation raises concerns about its reliability and validity. Nonetheless, the TSQ provided valuable preliminary insights, and further refinement through psychometric testing is necessary before its implementation in larger studies or clinical practice.

### Future research

Future research should aim to include larger and more diverse samples to enhance the generalizability of findings. Importantly, incorporating pre-treatment baseline assessments of cognitive, hearing, and olfactory functions would allow for a more accurate determination of chemotherapy-related changes over time. Including an age-matched control group without chemotherapy exposure would further strengthen causal interpretation. Expanding the study design to incorporate longitudinal follow-ups would allow for the assessment of both immediate and delayed effects of chemotherapy on hearing, olfactory function and cognitive performance. Tracking participants over time would enable the identification of changes occurring shortly after treatment as well as those developing over extended periods. Additionally, validating the TSQ could enhance the accuracy of olfactory function assessment and enable its integration into standardized test batteries.

## Conclusions

This pilot study demonstrated the feasibility of using a multifunctional test battery to assess sensory and cognitive effects of chemotherapy in cancer survivors. While no statistically significant differences were observed between the platinum- and taxane-based chemotherapy groups, differences in self-reported communication strategies suggest areas for further investigation. However, the lack of standardized data on chemotherapy dosage, session number, and time since treatment completion limits the interpretation of treatment-related effects. Future investigations should address these variables to clarify the relationship between treatment intensity and recovery patterns. Expanding the sample size and incorporating baseline and longitudinal follow-up assessments will be essential for validating these preliminary findings and improving generalizability. These results underscore the importance of comprehensive sensory and cognitive evaluations in cancer survivorship care. Further, large-scale studies are warranted to confirm these findings and refine assessment methodologies to optimize patient support and rehabilitation strategies.

## Supplementary Information

Supplementary Material 1.

## Data Availability

The datasets used and/or analysed during the current study are available from the corresponding author on reasonable request.

## Abbreviations

REC: Regional Committee for Medical and Health Research Ethics
DPO: Data Protection Officer
PTA4: Pure-tone average over 0.5, 1, 2, and 4 kHz
MoCA: Montreal Cognitive Assessment
HINT: Hearing in Noise Test
TMT: Trail Making Test
TEOAE: Transient Evoked Otoacoustic Emissions
DPOAE: Distortion Product Otoacoustic Emissions
TSQ: The Smelling Questionnaire
THI-NOR: Tinnitus Handicap Inventory (Norwegian version)
