# Supplementary material for: Hearing, smell, and cognitive function after cancer treatment
Source: BMC Cancer. 2025 Oct 1;25:1485. doi: 10.1186/s12885-025-14861-y (PMC12486504; doi:10.1186/s12885-025-14861-y)
Supplement: Supplementary file 1 — Supplementary Material 1. [file 12885_2025_14861_MOESM1_ESM.docx]

Appendix A. The Smelling Questionnaire

**Appendix A.** The Smelling Questionnaire (TSQ)

Appendix B. MoCA - Norwegian version and TMT A +B

**Appendix B** MoCA Norwegian version and TMT A + B


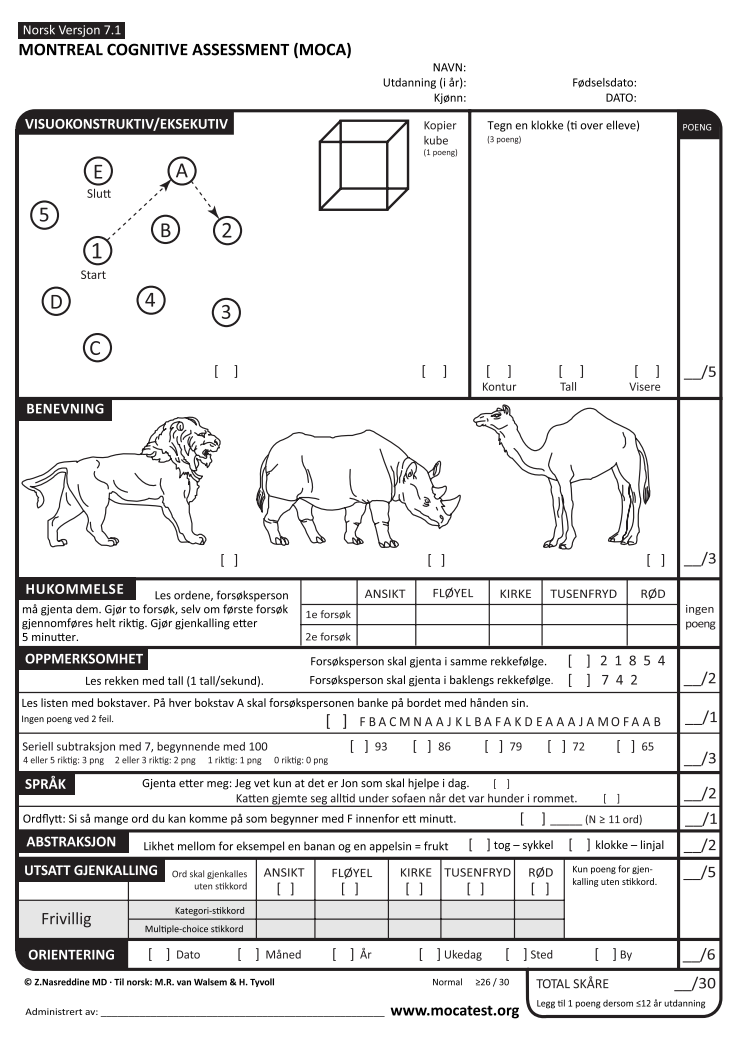


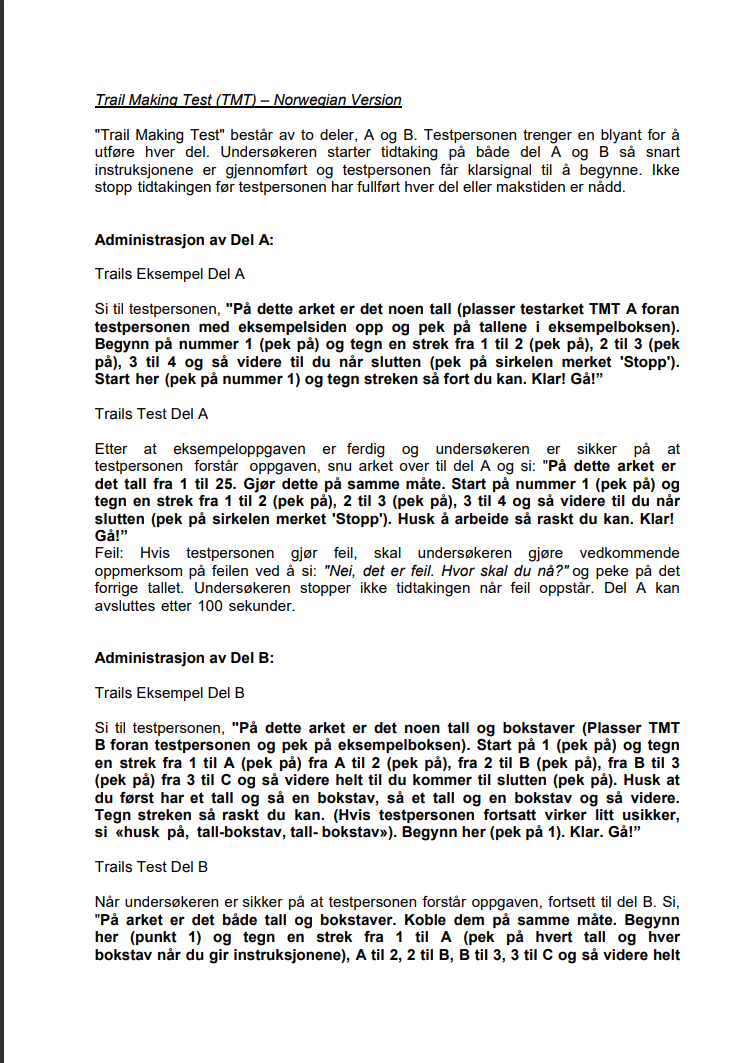


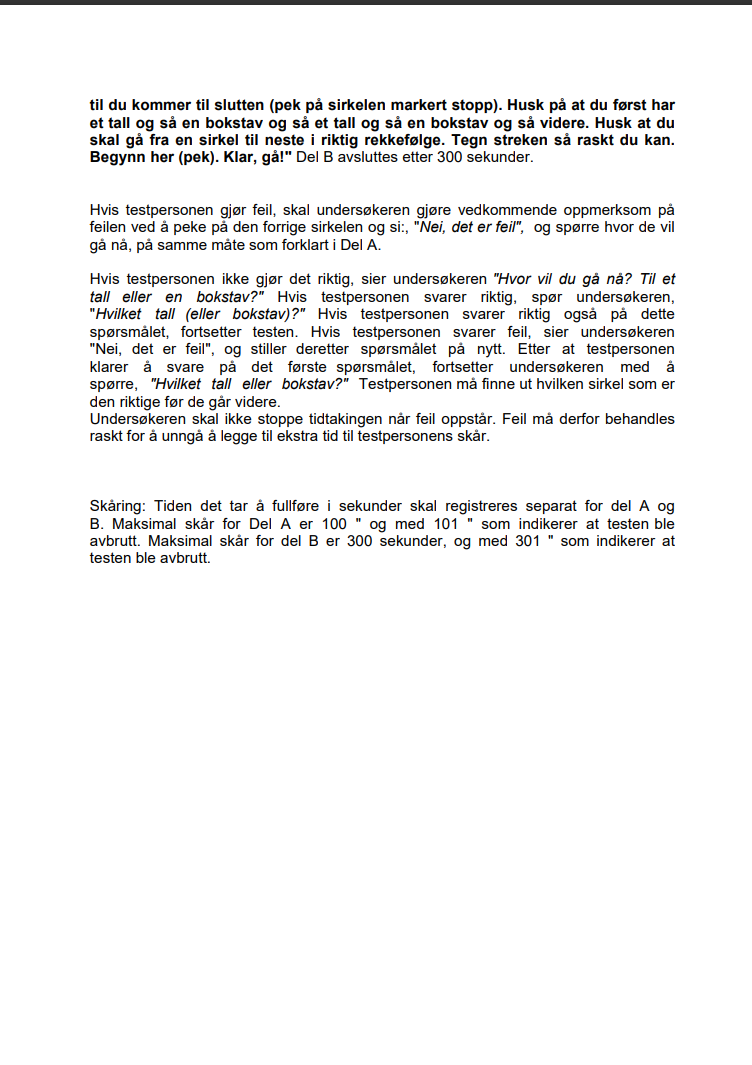


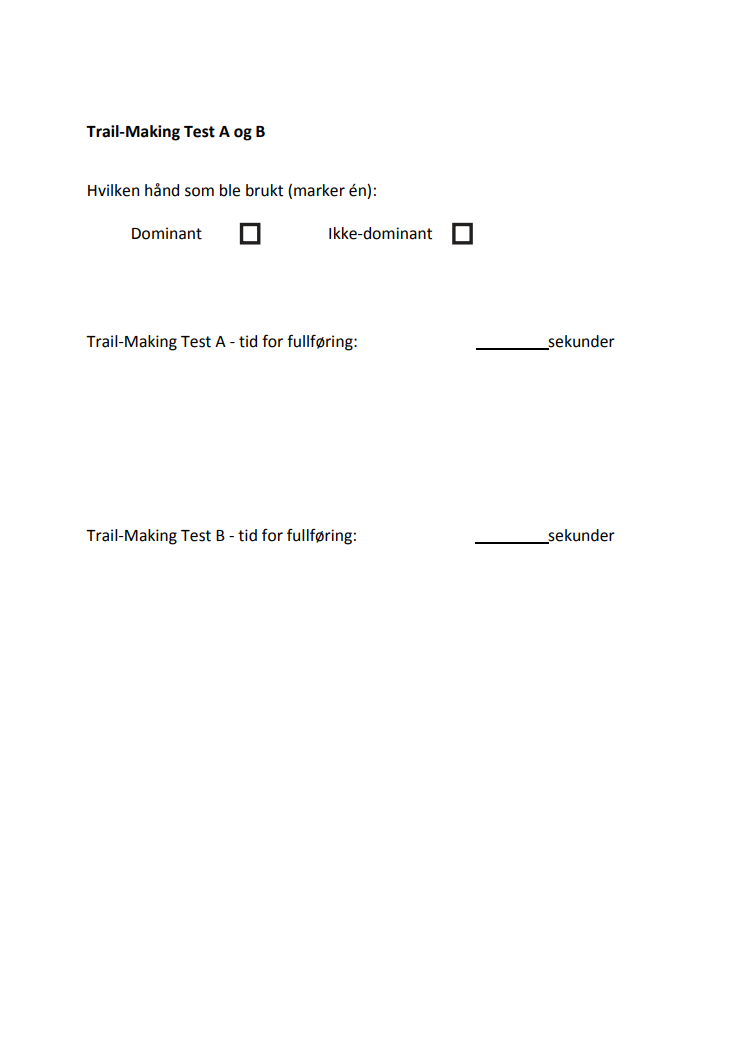


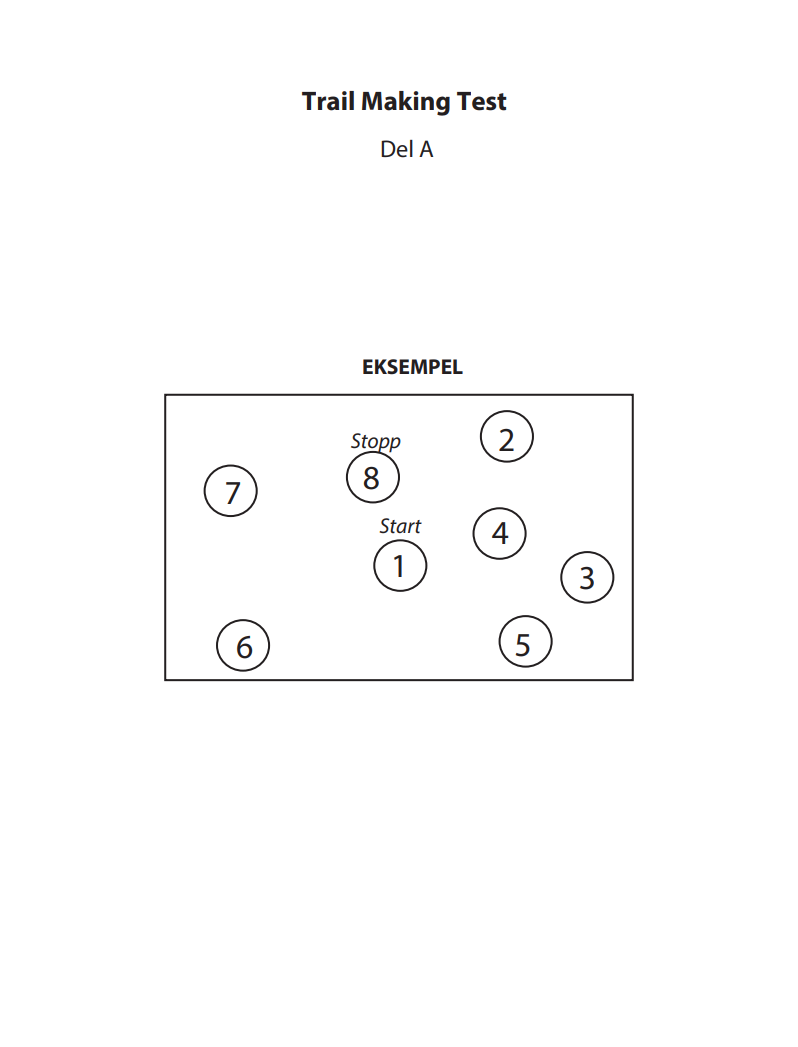


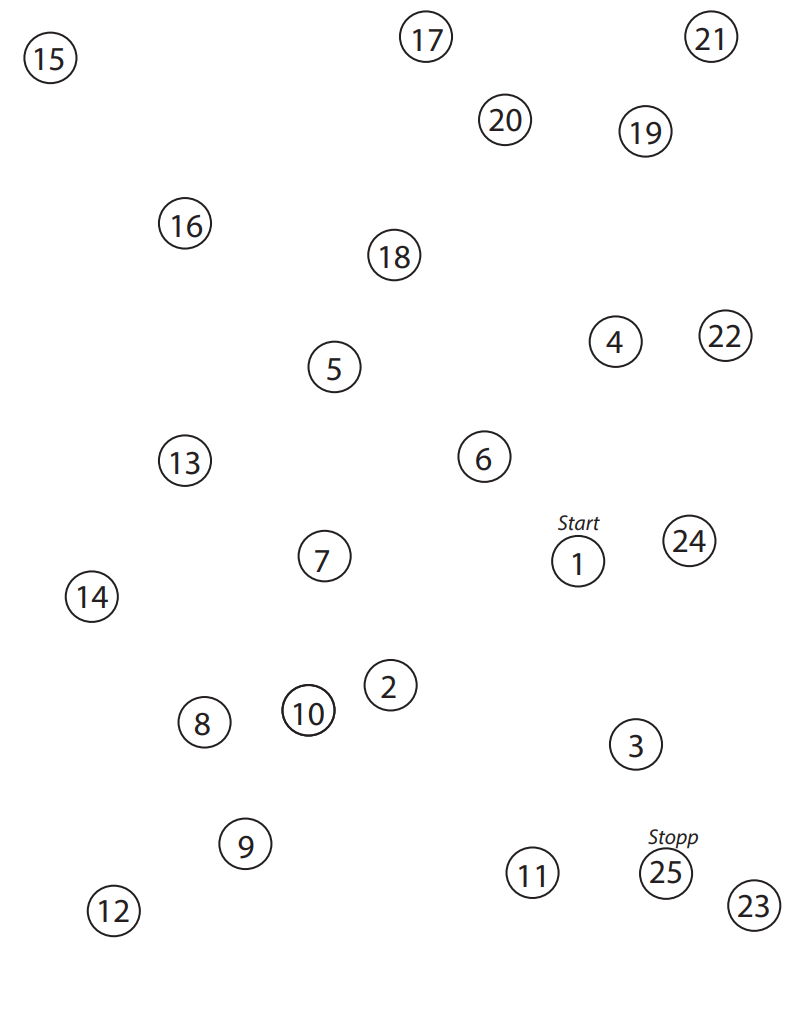


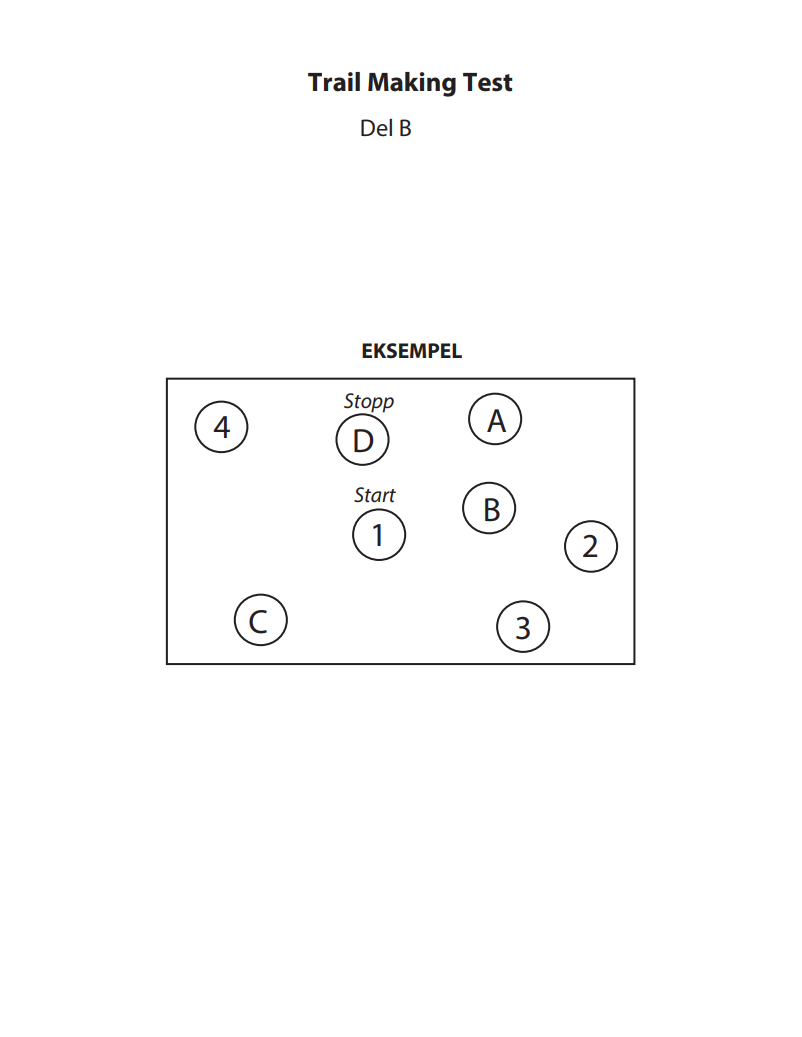


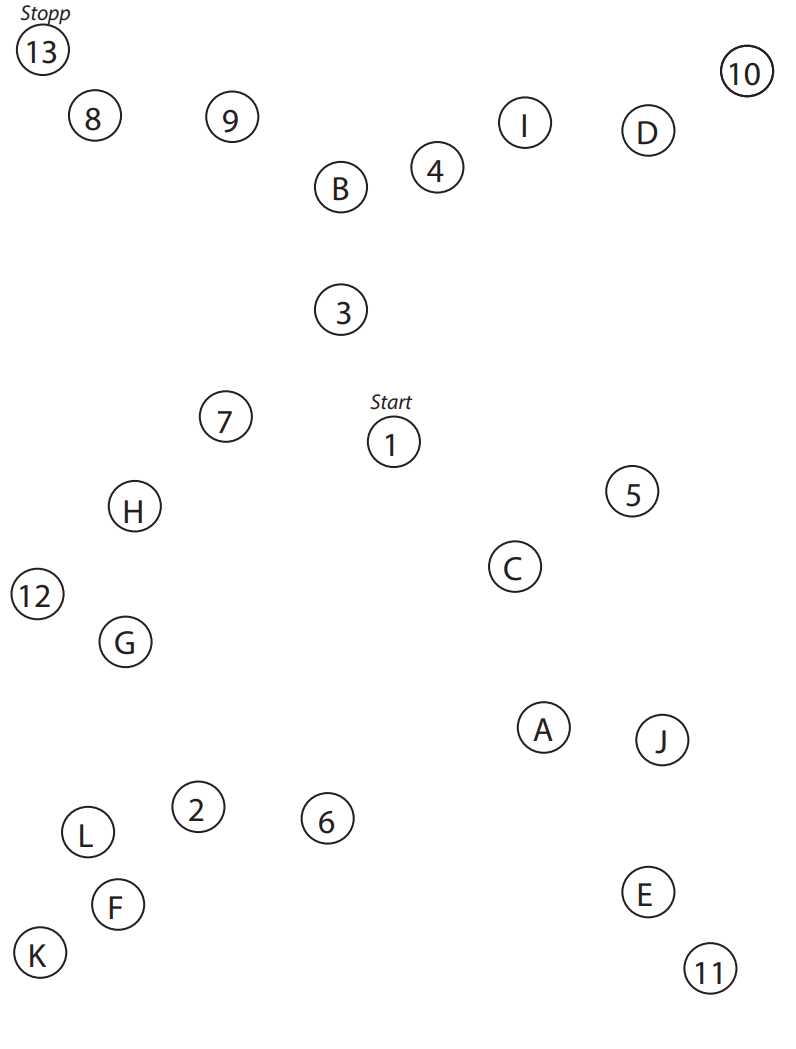


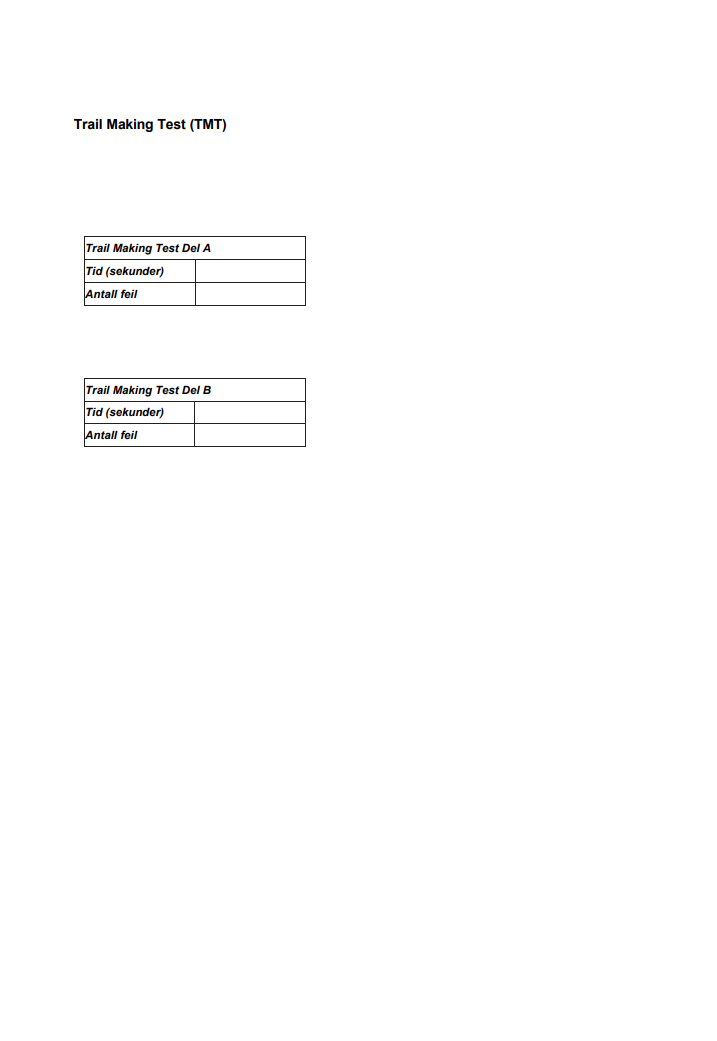


Appendix C. Communication Strategies Scale - Norwegian version

**Appendix C** The Norwegian version of Communication Strategies Scale (CSS)

Appendix D. Tinnitus Handicap Inventory (THI-NOR)

**Appendix D** Tinnitus Handicap Inventory (THI-NOR)

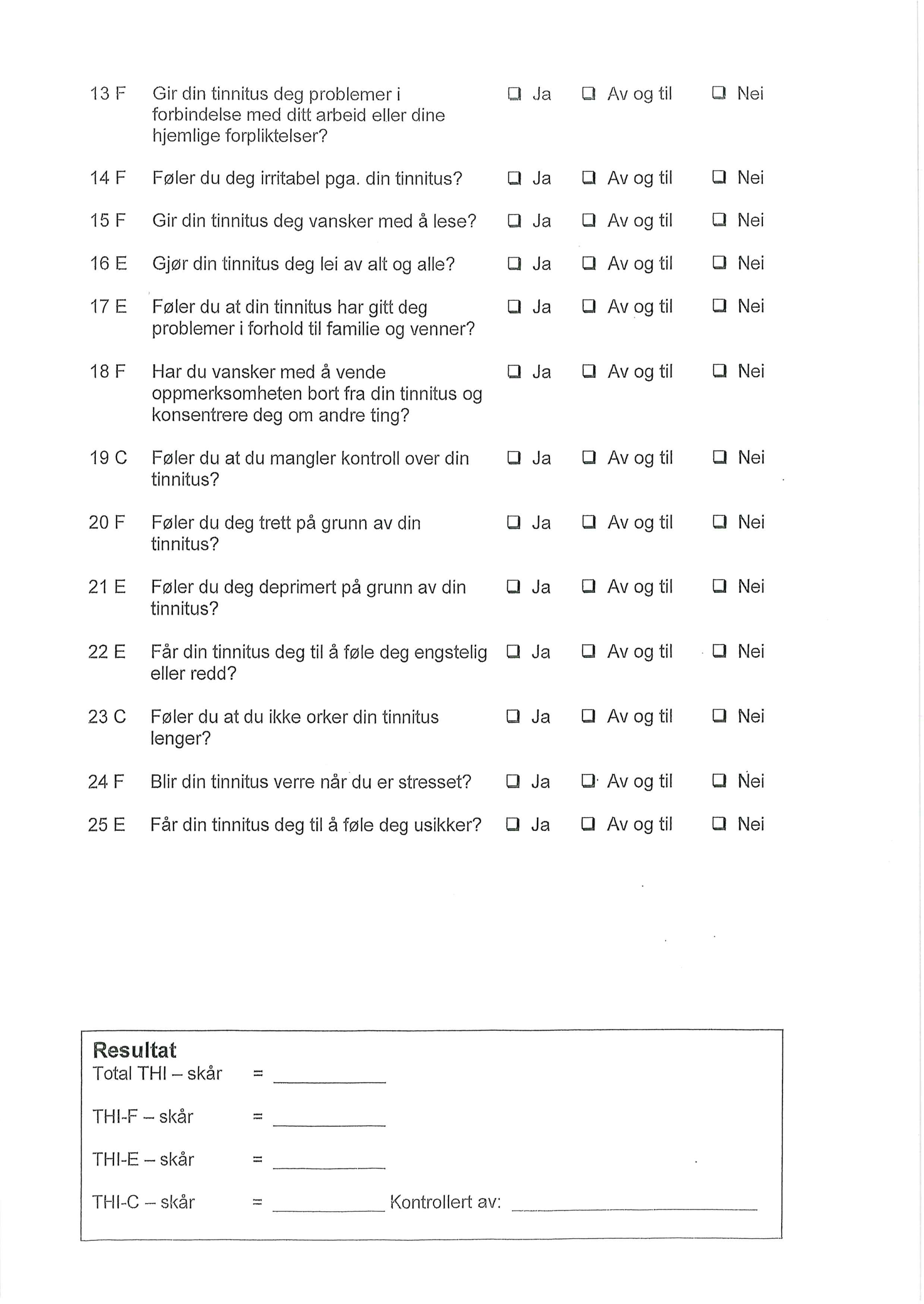


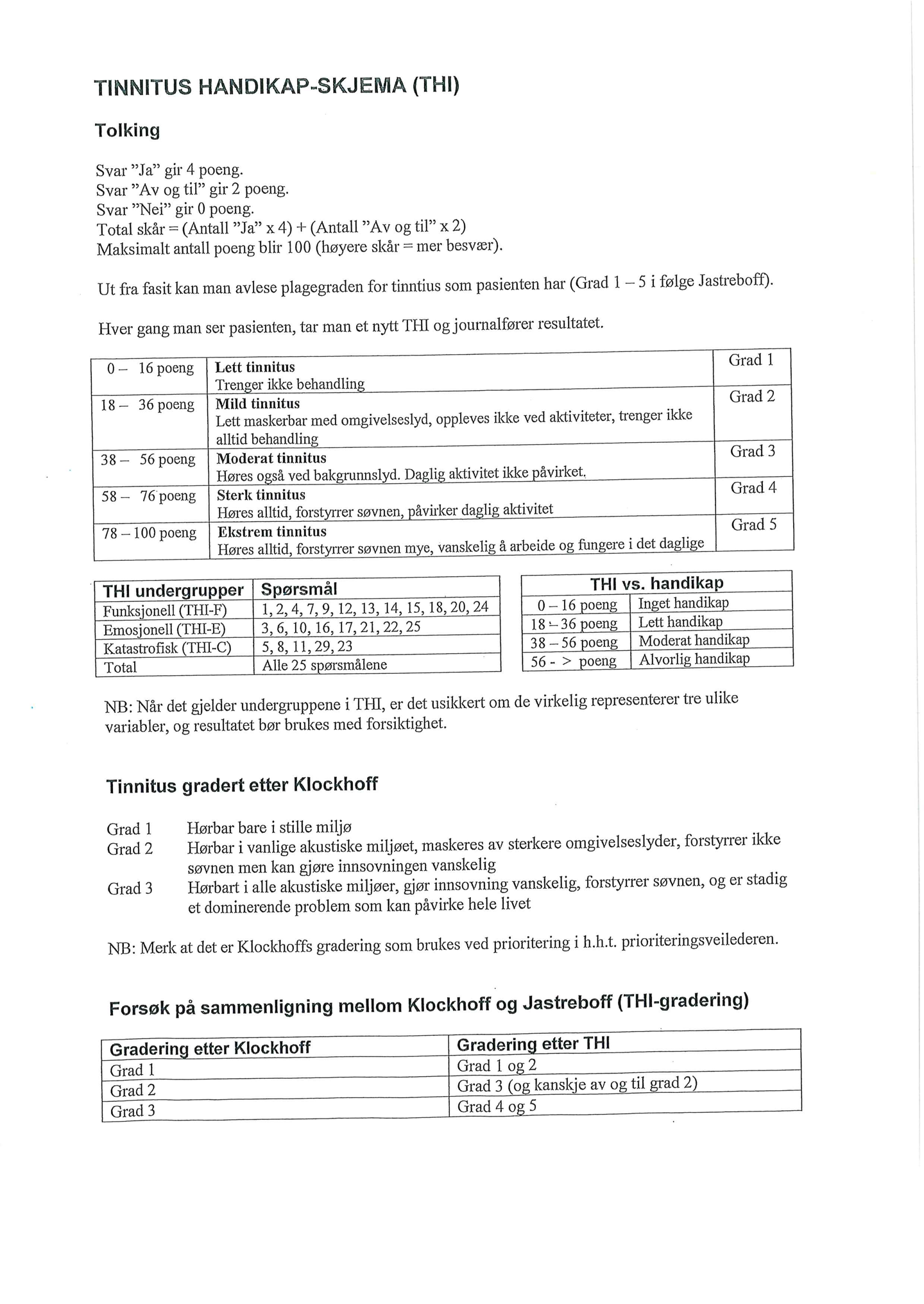


Appendix G. Consent Form

**Appendix E** Consent Form in Norwegian


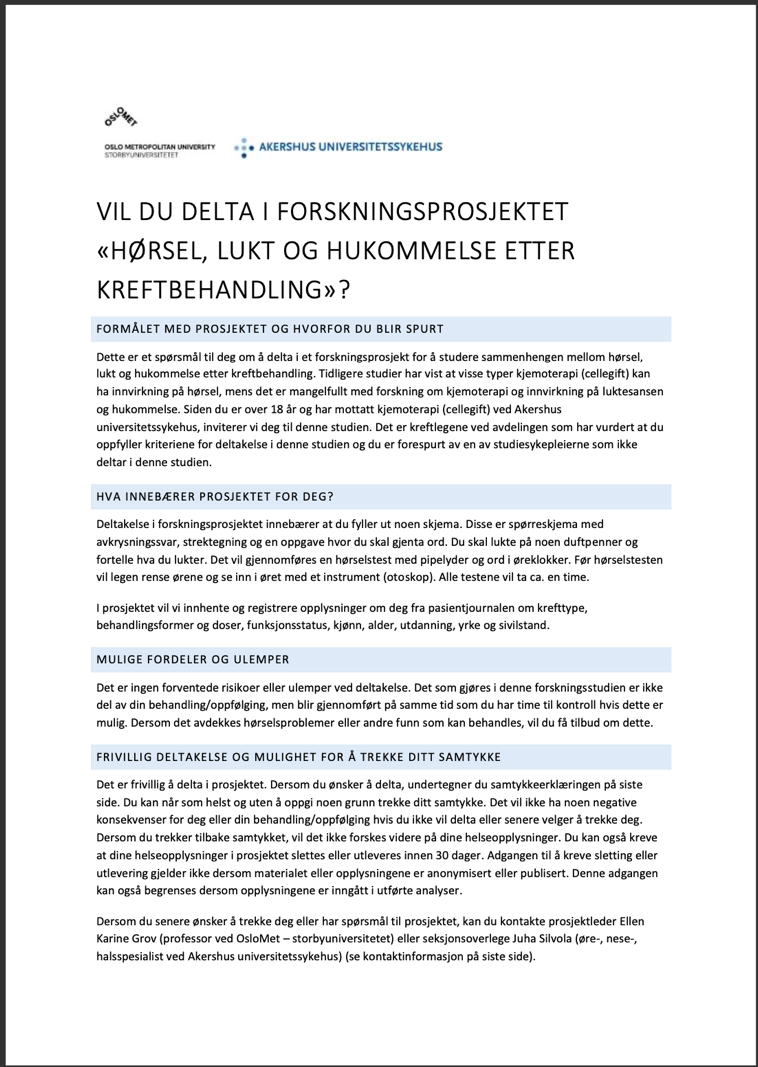


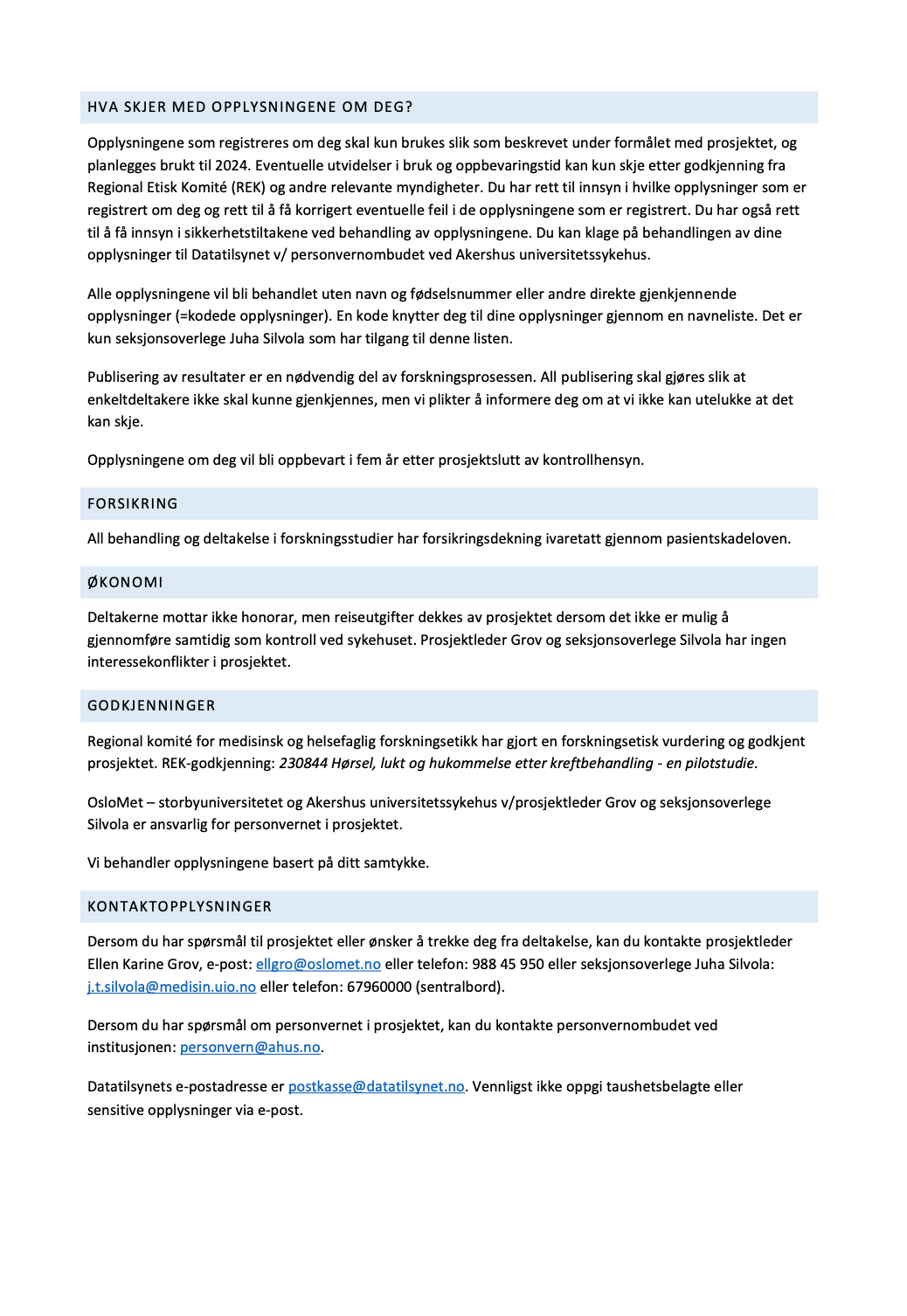


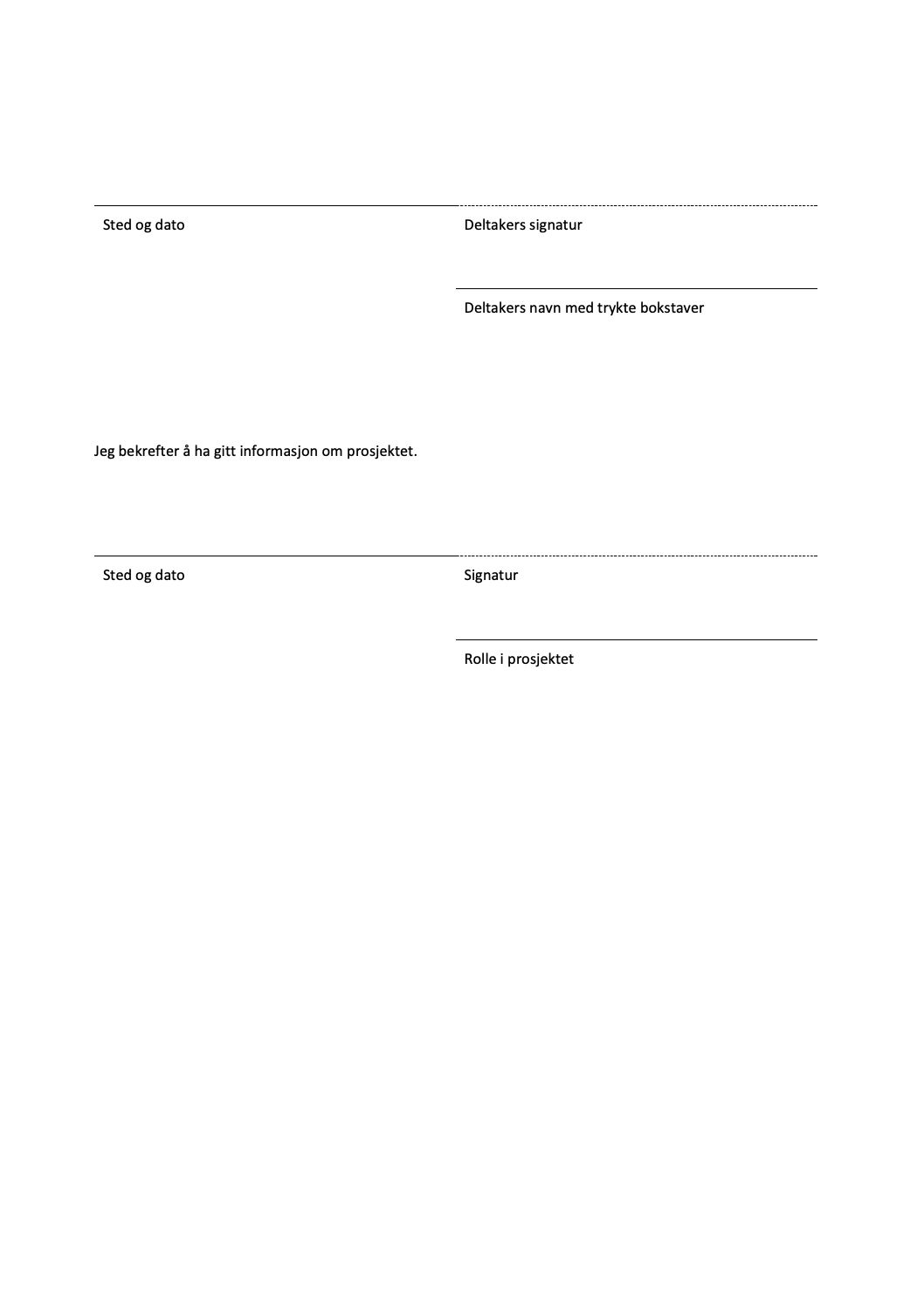


Appendix H. Tables

**Table H 1** Differences in TEOAE thresholds between Chemo 1 (P) and Chemo 2 (T)

| **TEOAE Test (Frequency)** | **Total Mean (*SD*)** | **Chemo 1 Mean (*SD*)** | **Chemo 2 Mean (*SD*)** | ***p*-value** | **CI 95% (Lower, Upper)** |
| --- | --- | --- | --- | --- | --- |
| TEOAE 1kHz Left | 0.92 (0.28) | 0.88 (0.35) | 1.00 (0.00) | 0.45 | -0.48, 0.23 |
| TEOAE 1.5kHz Left | 0.85 (0.38) | 0.88 (0.35) | 0.80 (0.45) | 0.74 | -0.41, 0.56 |
| TEOAE 2kHz Left | 0.77 (0.44) | 0.75 (0.46) | 0.80 (0.45) | 0.85 | -0.62, 0.52 |
| TEOAE 3kHz Left | 0.31 (0.48) | 0.25 (0.46) | 0.40 (0.55) | 0.61 | -0.77, 0.47 |
| TEOAE 4kHz Left | 0.15 (0.38) | 0.13 (0.35) | 0.20 (0.45) | 0.74 | -0.56, 0.41 |
| TEOAE 1kHz Right | 0.85 (0.38) | 0.75 (0.46) | 1.00 (0.00) | 0.26 | -0.71, 0.21 |
| TEOAE 1.5kHz Right | 0.77 (0.44) | 0.88 (0.35) | 0.60 (0.55) | 0.29 | -0.27, 0.82 |
| TEOAE 2kHz Right | 0.69 (0.48) | 0.75 (0.46) | 0.60 (0.55) | 0.61 | -0.47, 0.77 |
| TEOAE 3kHz Right | 0.38 (0.51) | 0.38 (0.52) | 0.40 (0.55) | 0.94 | -0.69, 0.64 |
| TEOAE 4kHz Right | 0.08 (0.28) | 0.13 (0.35) | 0.00 (0.00) | 0.45 | -0.23, 0.48 |

**Table H 2** Differences in DPOAE between Chemo 1 (P) and Chemo 2 (T)

| **DPOAE (Frequency)** | **Sample (*n*= 13) Mean (*SD*)** | | | **Chemo 1 (P) Mean (*SD*)** | **Chemo 2 (T) Mean (*SD*)** | | ***p*-value** | **CI 95% (Lower, Upper)** |
| --- | --- | --- | --- | --- | --- | --- | --- | --- |
| 0.5 kHz Left | | 0.23 (0.44) | | 0.00 (0.00) | | 0.60 (0.55) | 0.009 | -1.01, -0.19 |
| 1 kHz Left | | 0.54 (0.52) | | 0.38 (0.52) | | 0.80 (0.45) | 0.159 | -1.04, 0.19 |
| 1.5 kHz Left | | 0.69 (0.48) | | 0.75 (0.46) | | 0.60 (0.55) | 0.606 | -0.47, 0.77 |
| 2 kHz Left | | 0.31 (0.48) | | 0.38 (0.52) | | 0.20 (0.45) | 0.546 | -0.44, 0.79 |
| 3 kHz Left | | 0.23 (0.44) | | 0.25 (0.46) | | 0.20 (0.45) | 0.851 | -0.52, 0.62 |
| 4 kHz Left | | 0.38 (0.51) | | 0.38 (0.52) | | 0.40 (0.55) | 0.935 | -0.69, 0.64 |
| 6 kHz Left | | 0.00 (0.00) | | 0.00 (0.00) | | 0.00 (0.00) | 0.00 | 0.00, 0.00 |
| 8 kHz Left | | 0.00 (0.00) | | 0.00 (0.00) | | 0.00 (0.00) | 0.00 | 0.00, 0.00 |
| 10 kHz Left | | 0.00 (0.00) | | 0.00 (0.00) | | 0.00 (0.00) | 0.00 | 0.00, 0.00 |
| 0.5 kHz Right | | 0.69 (0.48) | 0.75 (0.46) | | | 0.60 (0.55) | 0.74 | -0.63, 0.41 |
| 1 kHz Right | | 54.00 (20.59) | 58.25 (22.05) | | | 47.20 (18.09) | 0.935 | -0.64, 0.69 |
| 1.5 kHz Right | | 60.62 (22.93) | 64.00 (24.40) | | | 55.20 (21.81) | 0.453 | -0.48, 0.23 |
| 2 kHz Right | | 64.31 (23.27) | 69.63 (22.01) | | | 55.80 (25.09) | 0.751 | -0.77, 0.58 |
| 3 kHz Right | | 64.54 (26.14) | 70.25 (27.11) | | | 55.40 (24.37) | 0.471 | -0.89, 0.44 |
| 4 kHz Right | | 69.00 (24.20) | 72.38 (28.49) | | | 63.60 (16.65) | 0.471 | -0.89, 0.44 |
| 5 kHz Right | | 76.69 (19.28) | 78.13 (24.01) | | | 74.40 (9.74) | 0.290 | -0.82, 0.27 |
| 6 kHz Right | | 72.38 (28.49) | 74.40 (16.65) | | | 69.00 (24.20) | 0.220 | -0.54, 0.14 |
| 7 kHz Right | | 70.25 (27.11) | 70.25 (27.11) | | | 63.60 (16.65) | 0.220 | -0.54, 0.14 |
| 8 kHz Right | | 0.00 (0.00) | 0.00 (0.00) | | | 0.20 (0.45) | 0.00 | 0.00, 0.00 |
| 9 kHz Right | | 0.00 (0.00) | 0.00 (0.00) | | | 0.00 (0.00) | 0.00 | 0.00, 0.00 |
| 10 kHz Right | | 0.00 (0.00) | 0.00 (0.00) | | | 0.00 (0.00) | 0.00 | 0.00, 0.00 |
|  | |  | |  | |  |  |  |

**Table H 3** Differences on speech recognition score on right and left ear between Chemo 1 (P) and Chemo 2 (T)

| **Speech Audiometry Score** | **Sample (n=13) Mean (*SD*)** | **Chemo 1 (P) Mean (*SD*)** | **Chemo 2 (T) Mean (*SD*)** | ***p*-value** | **CI 95% (Lower, Upper)** |
| --- | --- | --- | --- | --- | --- |
| Speech Audiometry Score (dB) Left | 53.09 (19.42) | 47.33 (23.59) | 60.00 (11.73) | 0.31 | -39.03, 13.70 |
| Speech Audiometry Score (dB) Right | 52.27 (16.18) | 50.00 (20.98) | 55.00 (9.35) | 0.64 | -28.06, 18.06 |
|  |  |  |  |  |  |

**Table H 4** Differences in odor recognition between Chemo 1 (P) and Chemo 2 (T)

| **Sniffing Test** | **Sample (n=13) Mean (*SD*)** | **Chemo 1 (P) Mean (*SD*)** | **Chemo 2 (T) Mean (*SD*)** | **p-value** | **CI 95% (Lower, Upper)** |
| --- | --- | --- | --- | --- | --- |
| Sniff Test 1 | 1.00 (0.00) | 1.00 (0.00) | 1.00 (0.00) | - | - |
| Sniff Test 2 | 0.85 (0.38) | 0.75 (0.46) | 1.00 (0.00) | 0.26 | -0.71, 0.21 |
| Sniff Test 3 | 0.92 (0.28) | 1.00 (0.00) | 0.80 (0.45) | 0.22 | -0.14, 0.54 |
| Sniff Test 4 | 0.92 (0.28) | 0.88 (0.35) | 1.00 (0.00) | 0.45 | -0.48, 0.23 |
| Sniff Test 5 | 0.85 (0.38) | 1.00 (0.00) | 0.60 (0.55) | 0.06 | -0.01, 0.81 |
| Sniff Test 6 | 1.00 (0.00) | 1.00 (0.00) | 1.00 (0.00) | - | - |
| Sniff Test 7 | 1.00 (0.00) | 1.00 (0.00) | 1.00 (0.00) | - | - |
| Sniff Test 8 | 0.85 (0.38) | 0.88 (0.35) | 0.80 (0.45) | 0.74 | -0.41, 0.56 |
| Sniff Test 9 | 0.85 (0.38) | 0.75 (0.46) | 1.00 (0.00) | 0.26 | -0.71, 0.21 |
| Sniff Test 10 | 0.92 (0.28) | 0.88 (0.35) | 1.00 (0.00) | 0.45 | -0.48, 0.23 |
| Sniff Test 11 | 0.77 (0.44) | 0.75 (0.46) | 0.80 (0.45) | 0.85 | -0.62, 0.52 |
| Sniff Test 12 | 0.92 (0.28) | 0.88 (0.35) | 1.00 (0.00) | 0.45 | -0.48, 0.23 |
| Sniff Test Total Score | 1.15 (1.28) | 1.25 (1.39) | 1.00 (1.22) | 0.75 | -1.42, 1.92 |

**Table H 5** Differences in Communication Strategies between Chemo 1 (P) and Chemo 2 (T)

| **CSS Questionnaire** | **Sample (n=13) Mean (*SD*)** | **Chemo 1 (P) Mean (*SD*)** | **Chemo 2 (T) Mean (*SD*)** | ***p*-value** | **CI 95% (Lower, Upper)** |
| --- | --- | --- | --- | --- | --- |
| Q1: Repeat | 2.53 (1.33) | 2.75 (1.49) | 2.20 (1.10) | 0.49 | -1.15, 2.25 |
| Q2: Interrupt | 2.53 (1.27) | 2.50 (1.41) | 2.60 (1.14) | 0.90 | -1.76, 1.56 |
| Q3: Dominate | 1.53 (1.13) | 1.75 (1.39) | 1.20 (0.45) | 0.42 | -0.88, 1.98 |
| Q4: Stop Repeat | 2.46 (1.39) | 2.75 (1.49) | 2.00 (1.22) | 0.37 | -1.00, 2.50 |
| Q5: Avoid Social | 1.84 (1.28) | 2.25 (1.49) | 1.20 (0.45) | 0.16 | -0.48, 2.58 |
| Q6: Avoid Conversing | 1.76 (1.30) | 2.12 (1.55) | 1.20 (0.45) | 0.23 | -0.67, 2.52 |
| Q7: Pretend | 2.15 (1.21) | 2.37 (1.41) | 1.80 (0.84) | 0.43 | -0.97, 2.12 |
| Q8: Avoid Strangers | 1.76 (1.36) | 2.00 (1.60) | 1.40 (0.89) | 0.46 | -1.14, 2.34 |
| Q9: Ignore | 2.07 (1.55) | 2.50 (1.85) | 1.40 (0.55) | 0.23 | -0.80, 3.00 |
| Q10: Ask Again | 3.23 (1.17) | 3.25 (1.28) | 3.20 (1.10) | 0.94 | -1.48, 1.58 |
| Q11: Repeat Part | 3.61 (1.50) | 3.75 (1.49) | 3.40 (1.67) | 0.70 | -1.60, 2.30 |
| Q12: Attention First | 2.07 (1.38) | 2.62 (1.51) | 1.20 (0.45) | 0.07 | -0.12, 2.97 |
| Q13: Ask for Repeat | 3.76 (1.36) | 4.25 (1.16) | 3.00 (1.41) | 0.11 | -0.33, 2.83 |
| Q14: Remind of Problem | 2.53(1.66) | 3.00 (1.93) | 1.80 (0.84) | 0.22 | -0.83, 3.23 |
| Q15: Attention from Friends | 1.53 (1.13) | 1.87 (1.36) | 1.00 (0.00) | 0.18 | -0.48, 2,23 |
| Q16: Explain Loss | 2.69 (1.65) | 3.12 (1.81) | 2.00 (1.22) | 0.25 | -0.91, 3,16 |
| Q17: Ask to Speak Up | 3.07 (1.26) | 3.37 (1.41) | 2.60 (0.89) | 0.30 | -0.79, 2.34 |
| Q18: Position for Hearing | 2.69 (1.38) | 3.37 (1.30) | 1.60 (0.55) | 0.02 | 0.41, 3.14 |
| Q19: Lip reading | 2.76(1.36) | 3.12 (1.46) | 2.20 (1.10) | 0.25 | -0.75, 2.60 |
| Q20: Relocate | 2.46 (1.05) | 2.75 (1.04) | 2.00 (1.00) | 0.22 | -0.53, 2,03 |
| Q21: Lighting | 2.30 (1.49) | 2.75 (1.67) | 1.60 (0.89) | 0.19 | -0.65, 2,95 |
| Q22: Positioning | 3.15 (1.41) | 3.75 (1.39) | 2.20 (0.84) | 0.05 | 0.02, 3.08 |
| Q23: Focus | 3.23 (1.24) | 3.87 (0.99) | 2.20 (0.84) | 0.01 | 0.50, 2.85 |
| Q24: Summarize | 3.23 (1.48) | 3.75 (1.28) | 2.40(1.52) | 0.11 | -0.37, 3.07 |
| Q25: Watch | 3.30 (1.55) | 3.87 (1.36) | 2.40 (1.52) | 0.10 | -0.30, 3.25 |

**Table H 6** THI DIfferences between Chemo 1 (P) and Chemo 2 (T)

| **THI Questionnaire** | **Sample (n=13) Mean (SD)** | **Chemo 1 (P) Mean (SD)** | **Chemo 2 (T) Mean (SD)** | ***p*-value** | **CI 95% (Lower, Upper)** |  |
| --- | --- | --- | --- | --- | --- | --- |
| **THI Q1:** | 2.31 (1.80) | 2.25 (1.67) | 2.40 (2.19) | 0.89 | -2.50, 2.20 | |
| **THI Q2:** | 1.23 (1.30) | 1.50 (1.41) | 0.80 (1.10) | 0.37 | -0.94, 2.34 | |
| **THI Q3:** | 0.15 (0.55) | 0.25 (0.71) | 0.00 (0.00) | 0.45 | -0.46, 0.96 | |
| **THI Q4:** | 1.08 (1.04) | 1.25 (1.04) | 0.80 (1.10) | 0.47 | -0.88, 1.78 | |
| **THI Q5:** | 1.08 (1.04) | 1.25 (1.04) | 0.80 (1.10) | 0.47 | -0.88, 1.78 | |
| **THI Q6:** | 0.15 (0.55) | 0.00 (0.00) | 0.40 (0.89) | 0.22 | -1.08, 0.28 | |
| **THI Q7:** | 1.69 (1.80) | 2.00 (1.85) | 1.20 (1.79) | 0.46 | -1.50, 3.10 | |
| **THI Q8:** | 1.85 (1.52) | 2.00 (1.51) | 1.60 (1.67) | 0.66 | -1.57, 2.37 | |
| **THI Q9:** | 1.69 (1.80) | 2.00 (1.85) | 1.20 (1.79) | 0.46 | -1.50, 3.10 | |
| **THI Q10:** | 1.23 (1.30) | 1.50 (1.41) | 0.80 (1.10) | 0.37 | -0.94, 2.34 | |
| **THI Q11:** | 0.00 (0.00) | 0.00 (0.00) | 0.00 (0.00) | - | -, - | |
| **THI Q12:** | 1.08 (1.04) | 1.25 (1.04) | 0.80 (1.10) | 0.47 | -0.88, 1.78 | |
| **THI Q13:** | 0.15 (0.55) | 0.25 (0.71) | 0.00 (0.00) | 0.45 | -0.46, 0.96 | |
| **THI Q14:** | 1.38 (1.26) | 1.50 (0.93) | 1.20 (1.79) | 0.70 | -1.34, 1.94 | |
| **THI Q15:** | 0.15 (0.55) | 0.25 (0.71) | 0.00 (0.00) | 0.45 | -0.46, 0.96 | |
| **THI Q16:** | 0.31 (0.75) | 0.25 (0.71) | 0.40 (0.89) | 0.74 | -1.13, 0.83 | |
| **THI Q17:** | 0.15 (0.55) | 0.25 (0.71) | 0.00 (0.00) | 0.45 | -0.46, 0.96 | |
| **THI Q18:** | 1.23 (1.30) | 1.50 (1.41) | 0.80 (1.10) | 0.37 | -0.94, 2.34 | |
| **THI Q19:** | 1.23 (1.01) | 1.50 (0.93) | 0.80 (1.10) | 0.24 | -0.54, 1.94 | |
| **THI Q20:** | 1.54 (1.66) | 1.50 (1.77) | 1.60 (1.67) | 0.92 | -2.28, 2.08 | |
| **THI Q21:** | 0.77 (1.01) | 1.00 (1.07) | 0.40 (0.89) | 0.32 | -0.67, 1.87 | |
| **THI Q22:** | 0.92 (1.04) | 1.00 (1.07) | 0.80 (1.10) | 0.75 | -1.15, 1.55 | |
| **THI Q23:** | 1.69 (1.38) | 1.75 (1.28) | 1.60 (1.67) | 0.86 | -1.65, 1.95 | |
| **THI Q24:** | 1.85 (1.72) | 2.00 (1.85) | 1.60 (1.67) | 0.70 | -1.84, 2.64 | |
| **THI Q25:** | 0.31 (0.75) | 0.25 (0.71) | 0.40 (0.89) | 0.74 | -1.13, 0.83 | |
| **Tinnitus Handicap Inventory Total Score** | 25.23 (21.86) | 28.25 (21.82) | 20.40 (23.51) | 0.55 | -20.32, 36.02 | |

**Table H 7** TSQ differences between Chemo 1 (P) and Chemo 2 (T)

| **TSQ Question** | **Sample (n=13) Mean (SD)** | **Chemo 1 (P) Mean (SD)** | **Chemo 2 (T) Mean (SD)** | ***p*-value** | **CI 95% (Lower, Upper)** |
| --- | --- | --- | --- | --- | --- |
| TSQ Q1 | 1.23 (0.44) | 1.25 (0.46) | 1.20 (0.45) | 0.85 | -0.52, 0.62 |
| TSQ Q2 | 0.69 (1.32) | 0.75 (1.39) | 0.60 (1.34) | 0.85 | -1.57, 1.87 |
| TSQ Q3 | 0.54 (1.05) | 0.63 (1.19) | 0.40 (0.89) | 0.72 | -1.14, 1.59 |
| TSQ Q4 | 1.54 (0.52) | 1.63 (0.52) | 1.40 (0.55) | 0.47 | -0.44, 0.89 |
| TSQ Q5 | 1.23 (1.42) | 1.00 (1.41) | 1.60 (1.52) | 0.48 | -2.42, 1.22 |
| TSQ Q6 | 1.08 (1.38) | 0.88 (1.25) | 1.40 (1.67) | 0.53 | -2.30, 1.25 |
| TSQ Q7 | 0.77 (0.93) | 0.63 (0.92) | 1.00 (1.00) | 0.50 | -1.56, 0.81 |
| TSQ Q8 | 0.62 (0.77) | 0.63 (0.92) | 0.60 (0.55) | 0.96 | -0.98, 1.03 |
| TSQ Q9 | 0.54 (0.66) | 0.50 (0.76) | 0.60 (0.55) | 0.80 | -0.96, 0.76 |
| TSQ Q10 | 0.62 (0.77) | 0.63 (0.92) | 0.60 (0.55) | 0.96 | -0.98, 1.03 |
